# Association of Cardiovascular Medications With Adverse Outcomes in a Matched Analysis of a National Cohort of Patients With COVID-19

**DOI:** 10.1016/j.ajmo.2023.100040

**Published:** 2023-03-22

**Authors:** Leonard K. Wang, Yong-Fang Kuo, Jordan Westra, Mukaila A. Raji, Mohanad Albayyaa, Joseph Allencherril, Jacques Baillargeon

**Affiliations:** a John Sealy School of Medicine, University of Texas Medical Branch, Galveston; b Department of Preventive Medicine and Population Health, University of Texas Medical Branch, Galveston; c Department of Internal Medicine, University of Texas Medical Branch, Galveston; d Institute for Translational Sciences, University of Texas Medical Branch; e Texas Heart Institute, Houston; f Section of Cardiology, Baylor College of Medicine, Houston, Texas

**Keywords:** Anticoagulants, COVID-19, Renin-angiotensin-aldosterone system blockers, Statins

## Abstract

**Background:**

The use of statins, angiotensin-converting enzyme inhibitors (ACEIs)/angiotensin II receptor blockers (ARBs), and anticoagulants may be associated with fewer adverse outcomes in COVID-19 patients.

**Methods:**

Nested within a cohort of 800,913 patients diagnosed with COVID-19 between April 1, 2020 and June 24, 2021 from the Optum COVID-19 database, three case-control studies were conducted. Cases—defined as persons who: (1) were hospitalized within 30 days of COVID-19 diagnosis (*n* = 88,405); (2) were admitted to the intensive care unit (ICU)/received mechanical ventilation during COVID-19 hospitalization (*n* = 22,147); and (3) died during COVID-19 hospitalization (*n* = 2300)—were matched 1:1 using demographic/clinical factors with controls randomly selected from a pool of patients who did not experience the case definition/event. Medication use was based on prescription ≤90 days before COVID-19 diagnosis.

**Results:**

Statin use was associated with decreased risk of hospitalization (adjusted odds ratio [aOR], 0.72; 95% confidence interval [95% CI], 0.69, 0.75) and ICU admission/mechanical ventilation (aOR, 0.90; 95% CI, 0.84, 0.97). ACEI/ARB use was associated with decreased risk of hospitalization (aOR, 0.67; 95% CI, 0.65, 0.70), ICU admission/mechanical ventilation (aOR, 0.92; 95% CI, 0.86, 0.99), and death (aOR, 0.60; 95% CI, 0.47, 0.78). Anticoagulant use was associated with decreased risk of hospitalization (aOR, 0.94; 95% CI, 0.89, 0.99) and death (aOR, 0.56; 95% CI, 0.41, 0.77). Interaction effects—in the model predicting hospitalization—were statistically significant for statins and ACEI/ARBs (*P* < .0001), statins and anticoagulants (*P* = .003), ACEI/ARBs and anticoagulants (*P* < .0001). An interaction effect—in the model predicting ventilator use/ICU—was statistically significant for statins and ACEI/ARBs (*P* = .002).

**Conclusions:**

Statins, ACEI/ARBs, and anticoagulants were associated with decreased risks of the adverse outcomes under study. These findings may provide clinically relevant information regarding potential treatment for patients with COVID-19.

## Background

As health care systems across the globe continue to battle the coronavirus disease 2019 (COVID-19) pandemic, identifying effective therapeutic options for the virus, particularly for patients with underlying comorbidities, is critically important.^1^, ^2^, ^3^ Biological pathways through which the severe acute respiratory syndrome coronavirus 2 (SARS-CoV-2) causes disease and mortality include systemic inflammation associated with a cytokine storm,^4^ immune dysregulation, and increased coagulopathy, associated with an increased risk of venous thromboembolism, stroke, and myocardial infarction.^5^^,^^6^ The use of existing drugs and their association with reduced COVID-19 disease severity and mortality is of great public health relevance. There is particular interest in examining the extent to which the use of three widely used cardiovascular medications—statins, renin-angiotensin-aldosterone system (RAAS) blockers, and anticoagulants—may be associated with reductions in COVID-19 morbidity and mortality.^1^^,^^7^

Statins, first-line lipid-lowering therapies, have consistently demonstrated potent antiinflammatory, antithrombotic, and immunomodulatory effects.^8^, ^9^, ^10^, ^11^ These pleiotropic effects may improve the clinical outcomes in patients with COVID-19 by reducing viral replication, endothelial dysfunction, and inflammatory dysregulation.^12^, ^13^, ^14^

Angiotensin-converting enzyme inhibitor (ACEI)/angiotensin II receptor blockers (ARBs), used as first-line hypertension therapies, have demonstrated antiinflammatory effects,^15^^,^^16^ specifically by blocking angiotensin-converting enzyme 2 (ACE2) downregulation-induced hyperactivation of the RAAS and attenuating oxidative stress, vasoconstriction, and inflammation.^7^^,^^17^ While there were initial theoretical concerns that ACEI/ARBs may increase the expression of the ACE2 receptor—the entry point for COVID-19 infection—thereby exacerbating COVID-19 infection susceptibility and disease progression,^7^^,^^18^ recent data from observational studies, randomized controlled trials, and metaanalyses have demonstrated that ACEI/ARBs do not have an adverse impact on clinical outcomes or prognosis of COVID-19 patients^17^^,^^19^, ^20^, ^21^, ^22^, ^23^, ^24^ and may, in fact, be associated with protective benefits.^7^^,^^25^^,^^26^

Anticoagulants are believed to mitigate the risk of microvascular and macrovascular thrombosis in COVID-19 patients.^27^^,^^28^ Anticoagulants such as unfractionated heparin have been shown to be efficacious and suppress the cytokine-mediated inflammatory process that results in endothelial dysfunction caused by COVID-19.^27^^,^^29^ Nevertheless, among the current data, controversy exists regarding the potential of anticoagulants to reduce adverse outcomes in COVID-19 patients.^29^, ^30^, ^31^, ^32^, ^33^ Our purpose was to examine the independent effect of each of these three classes of medication—statins, ACEI/ARBs, and anticoagulants—on COVID-19 outcomes in a large, demographically and clinically diverse cohort of COVID-19 patients.

## Methods

### Data Source

This series of nested case-control studies was based on Optum's longitudinal COVID-19 electronic health record (EHR) database, which was generated from the larger Optum EHR database to specifically study COVID-19. Including more than 90 million patients from 38 hospital networks and 18 nonnetwork hospitals in the United States, the database incorporates clinical and medical administrative data from both inpatient and ambulatory EHRs, practice management systems, and numerous other internal systems. Information was processed from across the continuum of care, including acute inpatient stays and outpatient visits. The database contains structured information, such as diagnosis, procedure codes, laboratory results, and clinical observations, including vital signs, blood pressure, pain, and body mass index (BMI). It also contains unstructured information in the form of clinical notes from office visits; consultation reports; discharge summaries; and reports from nursing records, pathology, radiology, and cardiology. Using a natural language processing (NLP) system, the unstructured data in the clinical notes are scanned to discover, interpret, and extract important clinical information.

The COVID-19 database captures point-of-care diagnostics specific to the COVID-19 patient during initial presentation, acute illness, and convalescence with over 500 mapped labs and bedside observations, including COVID-19-specific testing. The database includes all patients in the EHR database from January 2007 through June 24, 2021. The data were certified as deidentified by an independent statistical expert following Health Insurance Portability and Accountability Act (HIPAA) statistical deidentification rules and managed according to Optum® customer data use agreements. A data use agreement was developed with Optum, and this study was approved by the Institutional Review Board of the University of Texas Medical Branch at Galveston.

### COVID-19 Study Cohort

The study cohort is based on a cohort from the Optum COVID-19 database consisting of patients who were diagnosed with COVID-19 (by positive laboratory test (Appendix Table 4) or diagnosis [ICD-CM-10: U07.1]) between April 1, 2020, and June 24, 2021, and who received care in above-described health care delivery network, captured in the Optum EHR database, in the 12 months prior to COVID-19 diagnosis.

### Case Selection

Three nested case-control studies of COVID-19 outcomes were conducted (Table 1). For study 1, cases (*n* = 88,405) were defined as patients who were hospitalized within 30 days of their COVID-19 diagnosis that occurred 0-30 days following the COVID-19 diagnosis date. For study 2, cases (*n* = 22,147) were defined as patients who were admitted to the intensive care unit (ICU; had one encounter during the hospitalization that was coded as “Critical Care Unit [CCU]/Intensive Care Unit [ICU]”) or received mechanical ventilation (ICD-10-PCS: 5A09357, 5A09358, 5A09359, 5A0935A, 5A0935B, 5A0935Z, 5A09457, 5A09458, 5A09459, 5A0945A, 5A0945B, 5A0945Z, 5A09557, 5A09558, 5A09559, 5A0955A, 5A0955B, 5A0955Z, 5A19054, 5A1935Z, 5A1945Z, 5A1955Z; CPT: 94,002, 94,003, 94,004) during their COVID-19 hospitalization. For study 3, cases were defined as patients who died during their hospitalization for COVID-19 (*n* = 2300). Because the Optum EHR data only has month and year of death, along with a death indicator, patients needed to meet three criteria to be considered to have died during their hospitalization: (1) the patient had a positive death indicator; (2) the patient had a death month and year equal to the month and year of their hospitalization end; and (3) the patient had no encounters of any kind following the hospitalization end date.

**Table 1:** Cohort Flow Chart.

	Hospitalization		ICU admission/mechanical ventilation		Hospital mortality	
Step	Description	*N*	Description	*N*	Description	*N*
1	All patients in data	6,421,125	All patients in data	6,421,125	All patients in data	6,421,125
2	Patients in an integrated delivery network	5,704,773	Patients in an integrated delivery network	5704,773	Patients in an integrated delivery network	5704,773
3	Patients with complete demographic data	5,694,848	Patients with complete demographic data	5,694,848	Patients with complete demographic data	5,694,848
4	Patients with a COVID-19-positive lab test or diagnosis	800,913	Patients hospitalized within 30 days of diagnosis	87,758	Patients hospitalized within 30 days of diagnosis	87,758
4a	Patients hospitalized within 30 days of diagnosis	88,405	Patients with ventilation use or ICU stay in hospital	22,147	Patients who died in hospital	2328
4b	Patients not hospitalized	712,508	Patients with no ventilation/ICU	65,611	Patients who did not die	85,430
5	Patients matched	175,516	Patients matched	43,250	Patients matched	4600
5a	Cases	87,758	Cases	21,625	Cases	2300
5b	Controls	87,758	Controls	21,625	Controls	2300

### Control Selection

The underlying cohort from which controls were selected included patients in the Optum COVID-19 database who met the following criteria: were in an integrated delivery network, had complete demographic data, and had a positive COVID-19 lab test or diagnosis (Table 1). For study 1, controls (*n* = 87,758) were randomly selected from patients who were not hospitalized (*n* = 712,508). For study 2, controls (*n* = 21,625) were randomly selected from patients who were not admitted to the ICU or had mechanical ventilation (*n* = 65,611). For study 3, controls (*n* = 2300) were randomly selected from patients who did not die during their hospitalization (*n* = 85,430).

For each of the three case-control studies, cases were matched 1:1 with controls based on: sex, age (0-17, 18-39, 40-49, 50-59, 60-69, 70-79, ≥ 80), race (white, non-white), COPD (ICD-10-CM: I27.8x, I27.9, J41.x-J47.x, J60.x-J67.x, J68.4, J70.1, J70.3), obesity (ICD-10-CM: E66.x), atherosclerotic cardiovascular disease (ASCVD) (ICD-10-CM: I25.10), angina (ICD-10-CM: I20.x), diabetes (ICD-10-CM: E11.x-E13.x), hypertension (ICD-10-CM: I10.x-I15.x), and hyperlipidemia (ICD-10-CM: E78.x, E88.1).

### Exposure to CVD Medications

For each of the three CVD medication groups**—**statins, ACEI/ARBs, and anticoagulants**—**patients who received at least one prescription for the medication ≤90 days prior to their COVID-19 diagnosis date were defined as exposed.

### Covariates

For each case-control study, multivariable analyses were used to adjust for the following unmatched variables: Elixhauser comorbidity index score (with the six matched conditions removed)^34^ (0, 1, 2, ≥3), ethnicity (non-Hispanic, Hispanic), United States Census Bureau geographic region (Midwest, Northeast, South, West),^35^ receipt of remdesivir, and receipt of dexamethasone.

### Statistical Analysis

Patient demographic and clinical characteristics for the overall COVID-19 cohort and each matched case-control study were summarized using percentages. To compare the baseline covariates between groups, standardized differences were calculated^36^ in the cohort of COVID-19-positive patients based on three comparisons: hospitalization status, ventilation use or admission to ICU, and mortality.

For each nested matched case-control study, multivariable conditional logistic regression analysis, adjusting for each of the aforementioned unbalanced covariates, was used to calculate adjusted odds ratios (aORs) and 95% confidence intervals (95% CIs) for the three outcomes: hospitalization within 30 days of COVID-19 diagnosis, ICU admission/mechanical ventilation during COVID-19 hospitalization, and death during COVID-19 hospitalization.

Finally, to assess the robustness of our findings, several sensitivity analyses were conducted. First, for each medication group, analyses using prescription periods of ≤60 days and ≤120 days before the COVID-19 diagnosis date were assessed (Appendix Tables 1 and 2). Second, analyses were conducted in which patients who were in hospital networks with fewer than 100 COVID-19 patients (along with their matched pairs) were removed (Appendix Table 2). All analyses were performed using SAS version 9.4 (SAS Institute).

## Results

Table 2 presents the demographic and clinical characteristics of the overall COVID-19 study cohort and each of the three primary samples used in the case-control studies. For the ventilation/ICU status group, there were larger standardized differences^37^ in in-hospital dexamethasone and remdesivir use. For the hospitalization status group, there was a larger standardized difference^37^ in the Elixhauser comorbidity index.

**Table 2:** Patient Characteristics by Hospitalization, ICU Admission/Mechanical Ventilation, and Hospital Mortality.

		Hospitalization status			Ventilation or ICU status			Death status		
Parameter	Level	Cases *N* (Column%)	Controls *N* (Column%)	Standardized difference*	Cases *N* (Column%)	Controls *N* (Column%)	Standardized difference*	Cases *N* (Column%)	Controls *N* (Column%)	Standardized difference*
Total		87,758	87,758		21,625	21,625		2300	2300	
Dexamethasone Use In-Hospital	No				10,069 (46.6%)	12,707 (58.8%)	0.2462	1082 (47.0%)	1304 (56.7%)	0.4805
	Yes				11,556 (53.4%)	8918 (41.2%)		1218 (53.0%)	996 (43.3%)	
Remdesivir Use In-Hospital	No				18,848 (87.2%)	19,919 (92.1%)	0.163	1756 (76.3%)	2076 (90.3%)	0.4482
	Yes				2777 (12.8%)	1706 (7.9%)		544 (23.7%)	224 (9.7%)	
Ethnicity	Non-Hispanic	70,790 (80.7%)	71,460 (81.4%)	0.1388	17,398 (80.5%)	17,639 (81.6%)	0.0519	1839 (80.0%)	1901 (82.7%)	0.0841
	Hispanic	10,453 (11.9%)	8214 (9.4%)		2549 (11.8%)	2440 (11.3%)		247 (10.7%)	238 (10.3%)	
	Unknown	6515 (7.4%)	8084 (9.2%)		1678 (7.8%)	1546 (7.1%)		214 (9.3%)	161 (7.0%)	
Region	Midwest	39,564 (45.1%)	42,363 (48.3%)	0.1441	10,182 (47.1%)	9385 (43.4%)	0.1672	823 (35.8%)	1031 (44.8%)	0.3671
	Northeast	21,801 (24.8%)	24,282 (27.7%)		4414 (20.4%)	5840 (27.0%)		448 (19.5%)	588 (25.6%)	
	Other/ Unknown	2796 (3.2%)	2894 (3.3%)		606 (2.8%)	654 (3.0%)		54 (2.3%)	79 (3.4%)	
	South	18,395 (21.0%)	13,614 (15.5%)		5004 (23.1%)	4475 (20.7%)		789 (34.3%)	456 (19.8%)	
	West	5202 (5.9%)	4605 (5.2%)		1419 (6.6%)	1271 (5.9%)		186 (8.1%)	146 (6.3%)	
Elixhauser Category	0	21,824 (24.9%)	36,281 (41.3%)	0.3922	3406 (15.8%)	4751 (22.0%)	0.2908	454 (19.7%)	438 (19.0%)	0.1579
	1	16,789 (19.1%)	17,874 (20.4%)		3090 (14.3%)	4334 (20.0%)		292 (12.7%)	389 (16.9%)	
	2	13,103 (14.9%)	11,111 (12.7%)		3106 (14.4%)	3476 (16.1%)		267 (11.6%)	334 (14.5%)	
	3+	36,042 (41.1%)	22,492 (25.6%)		12,023 (55.6%)	9064 (41.9%)		1287 (56.0%)	1139 (49.5%)	
Gender	Female	45,138 (51.4%)	45,138 (51.4%)	0	9000 (41.6%)	9000 (41.6%)	0	932 (40.5%)	932 (40.5%)	0
	Male	42,620 (48.6%)	42,620 (48.6%)		12,625 (58.4%)	12,625 (58.4%)		1368 (59.5%)	1368 (59.5%)	
Age Category	Mean (SD)	59.7 (19.2)	59.4 (19.3)	0.0161	63.2 (16.4)	63.3 (16.5)	−0.0024	71.3 (12.8)	70.9 (13.1)	0.0326
	<18	1235 (1.4%)	1235 (1.4%)		238 (1.1%)	238 (1.1%)		3 (0.1%)	3 (0.1%)	
	18-39	14,620 (16.7%)	14,620 (16.7%)		1723 (8.0%)	1723 (8.0%)		39 (1.7%)	39 (1.7%)	
	40-49	8537 (9.7%)	8537 (9.7%)		1950 (9.0%)	1950 (9.0%)		91 (4.0%)	91 (4.0%)	
	50-59	14,172 (16.1%)	14,172 (16.1%)		3725 (17.2%)	3725 (17.2%)		257 (11.2%)	257 (11.2%)	
	60-69	18,625 (21.2%)	18,625 (21.2%)		5699 (26.4%)	5699 (26.4%)		550 (23.9%)	550 (23.9%)	
	70-79	16,246 (18.5%)	16,246 (18.5%)		4848 (22.4%)	4848 (22.4%)		679 (29.5%)	679 (29.5%)	
	80+	14,323 (16.3%)	14,323 (16.3%)		3442 (15.9%)	3442 (15.9%)		681 (29.6%)	681 (29.6%)	
Race	African American	15,640 (17.8%)	14,934 (17.0%)	0.0863	3802 (17.6%)	4015 (18.6%)	0.0366	353 (15.3%)	384 (16.7%)	0.0747
	Asian	2286 (2.6%)	1907 (2.2%)		609 (2.8%)	556 (2.6%)		76 (3.3%)	69 (3.0%)	
	Caucasian	57,899 (66.0%)	57,899 (66.0%)		14,177 (65.6%)	14,177 (65.6%)		1564 (68.0%)	1564 (68.0%)	
	Other/ Unknown	11,933 (13.6%)	13,018 (14.8%)		3037 (14.0%)	2877 (13.3%)		307 (13.3%)	283 (12.3%)	
COPD		22,192 (25.3%)	22,192 (25.3%)	0	6267 (29.0%)	6267 (29.0%)	0	640 (27.8%)	640 (27.8%)	0
Obesity		26,748 (30.5%)	26,748 (30.5%)	0	7540 (34.9%)	7540 (34.9%)	0	706 (30.7%)	706 (30.7%)	0
Atherosclerotic Cardiovascular Disease		15,785 (18.0%)	15,785 (18.0%)	0	4645 (21.5%)	4645 (21.5%)	0	604 (26.3%)	604 (26.3%)	0
Angina		1932 (2.2%)	1932 (2.2%)	0	447 (2.1%)	447 (2.1%)	0	39 (1.7%)	39 (1.7%)	0
Diabetes		29,032 (33.1%)	29,032 (33.1%)	0	8971 (41.5%)	8971 (41.5%)	0	919 (40.0%)	919 (40.0%)	0
Hyperlipidemia		37,848 (43.1%)	37,848 (43.1%)	0	10,765 (49.8%)	10,765 (49.8%)	0	1145 (49.8%)	1145 (49.8%)	0
Hypertension		50,827 (57.9%)	50,827 (57.9%)	0	14,672 (67.8%)	14,672 (67.8%)	0	1537 (66.8%)	1537 (66.8%)	0

⁎ Standardized differences were calculated using SAS version 9.4 (SAS Institute, Cary, North Carolina).^36^

Table 3 presents the results for each of the 3 case-control studies. For study 1, the multivariable conditional logistic regression showed that use of statins (aOR, 0.72; 95% CI, 0.69, 0.75), ACEI/ARBs (aOR, 0.67; 95% CI, 0.65, 0.70), and anticoagulants (aOR, 0.94; 95% CI, 0.89, 0.99) was associated with a decreased risk of hospitalization. For study 2, the multivariable conditional logistic regression showed that use of statins (aOR, 0.90; 95% CI, 0.84, 0.97) and ACEI/ARBs (aOR, 0.92; 95% CI, 0.86, 0.99) was associated with a decreased risk in ICU admission/mechanical ventilation. For study 3, the conditional logistic regression showed that use of both ACEI/ARBs (aOR, 0.60; 95% CI, 0.47, 0.78) and anticoagulants (aOR, 0.56; 95% CI, 0.41, 0.77) was associated with a decreased risk of hospital mortality.

**Table 3:** Unadjusted and Adjusted Odds Ratios for Hospitalization, ICU Admission/Mechanical Ventilation, and Hospital Mortality Due to COVID-19.⁎

Characteristic	Case no. (%)		Control⁎⁎ no. (%)	Unadjusted OR† (95% CI‡)		Adjusted OR (95% CI)
Case-control study predicting hospitalization						
Statin Use	7875 (9.0%)		11,607 (13.2%)	0.61 (0.59, 0.63)		0.72 (0.69, 0.75)
ACEI/ARB Use	7690 (8.8%)		12,195 (13.9%)	0.56 (0.55, 0.58)		0.67 (0.65, 0.70)
Anticoagulant Use	3822 (4.4%)		3558 (4.1%)	1.08 (1.03, 1.13)		0.94 (0.89, 0.99)
Case-control study predicting ICU admission/mechanical ventilation						
Statin Use	2111 (9.8%)		2380 (11.0%)	0.86 (0.81, 0.92)		0.90 (0.84, 0.97)
ACEI/ARB Use	2051 (9.5%)		2289 (10.6%)	0.88 (0.82, 0.94)		0.92 (0.86, 0.99)
Anticoagulant Use	1064 (4.9%)		1014 (4.7%)	1.05 (0.96, 1.15)		0.96 (0.88, 1.06)
Case-control study predicting in-hospital mortality						
Statin Use		164 (7.1%)	233 (10.1%)	0.66 (0.53, 0.82)	0.84 (0.65, 1.07)	
ACEI/ARB Use		152 (6.6%)	238 (10.3%)	0.60 (0.48, 0.74)	0.60 (0.47, 0.78)	
Anticoagulant Use		75 (3.3%)	135 (5.9%)	0.55 (0.41, 0.73)	0.56 (0.41, 0.77)	

⁎ Multivariable analyses were adjusted for all unmatched variables including statin use, ACEI/ARB use, anticoagulant use, ethnicity, region, and Elixhauser score.

⁎⁎ Each case-control study was matched on sex, race, age group, and each of the following medical conditions: COPD, obesity, cardiovascular disease, angina, diabetes, hyperlipidemia, and hypertension.

† OR, odds ratio.

‡ 95% CI, 95% confidence interval.

Two-way interaction effects—assessed in the multivariable model predicting hospitalization—were statistically significant for: statins and ACEI/ARBs (*P* < .0001), statins and anticoagulants (*P* = .003), and ACEI/ARBs and anticoagulants (*P* < .0001). Likewise, a two-way drug interaction effect—assessed in the model predicting ICU admission/mechanical ventilation—was statistically significant for statins and ACEI/ARBs (*P* = .002). Stratified analysis showed that use of ACEI/ARBs was protective against hospitalization in persons who were not concomitantly taking statins (odds ratio [OR], 0.56; 95% CI, 0.54, 0.59) compared to those who were (OR, 0.97; 95% CI, 0.85, 1.11) or in persons who were not concomitantly taking anticoagulants (OR, 0.65; 95% CI, 0.62, 0.67) compared to those who were (OR, 1.01; 95% CI, 0.69, 1.48); and statins were protective against hospitalization in persons who were not concomitantly taking ACEI/ARBs (OR, 0.63; 95% CI, 0.60, 0.66) compared to those who were (OR, 0.95; 95% CI, 0.82, 1.11).

To assess the robustness of our findings, we conducted a number of sensitivity analyses. First, logistic regression models using prescription periods of ≤60 days and ≤120 days before the COVID-19 diagnosis date were assessed (Appendix Tables 1 and 2). Next, logistic regression models in which patients who were in hospital networks with ≤100 COVID-19 patients (along with their matched pairs) were removed (Appendix Table 3) were conducted. In each of the above sensitivity analyses, the direction and magnitude of the ORs for each of the three medications under study were consistent with the overall study findings.

**Appendix Table 1:** Sensitivity Analyses Based on Prescription Period of ≤60 Days.⁎

Characteristic	Case no. (%)		Control⁎⁎ no. (%)	Unadjusted OR† (95% CI‡)		Adjusted OR (95% CI)
Case-control study predicting hospitalization						
Statin Use	5748 (6.6%)		8565 (9.8%)	0.62 (0.60, 0.65)		0.72 (0.69, 0.75)
ACEI/ARB Use	5639 (6.4%)		9137 (10.4%)	0.57 (0.55, 0.59)		0.67 (0.64, 0.70)
Anticoagulant Use	3035 (3.5%)		2758 (3.1%)	1.11 (1.05, 1.17)		0.95 (0.90, 1.01)
Case-control study predicting ICU admission/mechanical ventilation						
Statin Use	1566 (7.2%)		1741 (8.1%)	0.88 (0.82, 0.95)		0.93 (0.86, 1.01)
ACEI/ARB Use	1480 (6.8%)		1693 (7.8%)	0.86 (0.80, 0.93)		0.88 (0.81, 0.96)
Anticoagulant Use	817 (3.8%)		805 (3.7%)	1.02 (0.92, 1.12)		0.93 (0.84, 1.04)
Case-control study predicting hospital mortality						
Statin Use		123 (5.4%)	170 (7.4%)	0.69 (0.54, 0.89)	0.89 (0.67, 1.18)	
ACEI/ARB Use		99 (4.3%)	172 (7.5%)	0.55 (0.42, 0.71)	0.58 (0.43, 0.77)	
Anticoagulant Use		56 (2.4%)	103 (4.5%)	0.53 (0.38, 0.74)	0.52 (0.36, 0.75)	

⁎ Multivariable analyses were adjusted for all unmatched variables including statin use, ACEI/ARB use, anticoagulant use, ethnicity, region, and Elixhauser score.

⁎⁎ Each case-control study was matched on sex, race, age group, and each of the following medical conditions: COPD, obesity, cardiovascular disease, angina, diabetes, hyperlipidemia, and hypertension.

† OR, odds ratio.

‡ 95% CI, 95% confidence interval.

**Appendix Table 2:** Sensitivity Analyses Based on Prescription Period of ≤120 Days.⁎

Characteristic	Case no. (%)		Control⁎⁎ no. (%)	Unadjusted OR† (95% CI‡)		Adjusted OR (95% CI)
Case-control study predicting hospitalization						
Statin Use	9395 (10.7%)		13,824 (15.8%)	0.60 (0.58, 0.61)		0.71 (0.69, 0.74)
ACEI/ARB Use	9184 (10.5%)		14,488 (16.5%)	0.55 (0.53, 0.57)		0.66 (0.64, 0.69)
Anticoagulant Use	4369 (5.0%)		4159 (4.7%)	1.06 (1.01, 1.10)		0.92 (0.88, 0.96)
Case-control study predicting ICU admission/mechanical ventilation						
Statin Use	2542 (11.8%)		2837 (13.1%)	0.87 (0.82, 0.92)		0.91 (0.85, 0.98)
ACEI/ARB Use	2486 (11.5%)		2734 (12.6%)	0.89 (0.84, 0.95)		0.92 (0.86, 0.99)
Anticoagulant Use	1210 (5.6%)		1164 (5.4%)	1.04 (0.96, 1.13)		0.94 (0.86, 1.03)
Case-control study predicting hospital mortality						
Statin Use		208 (9.0%)	284 (12.3%)	0.68 (0.56, 0.83)	0.86 (0.68, 1.08)	
ACEI/ARB Use		196 (8.5%)	292 (12.7%)	0.63 (0.52, 0.76)	0.64 (0.51, 0.81)	
Anticoagulant Use		94 (4.1%)	165 (7.2%)	0.53 (0.41, 0.70)	0.54 (0.40, 0.73)	

⁎ Multivariable analyses were adjusted for all unmatched variables including statin use, ACEI/ARB use, anticoagulant use, ethnicity, region, and Elixhauser score.

⁎⁎ Each case-control study was matched on sex, race, age group, and each of the following medical conditions: COPD, obesity, cardiovascular disease, angina, diabetes, hyperlipidemia, and hypertension.

† OR, odds ratio.

‡ 95% CI, 95% confidence interval.

**Appendix Table 3:** Sensitivity Analyses Restricted to Patients From Health Systems With ≥100 COVID-19 Patients With Hospitalization and ≥100 COVID-19 Patients With ICU Admission/Mechanical Ventilation and Hospital Mortality.⁎

Characteristic	Case no. (%)		Control⁎⁎ no. (%)	Unadjusted OR† (95% CI‡)		Adjusted OR (95% CI)
Case-control study predicting hospitalization						
Statin Use	7870 (9.0%)		11,606 (13.2%)	0.61 (0.59, 0.63)		0.72 (0.69, 0.75)
ACEI/ARB Use	7690 (8.8%)		12,195 (13.9%)	0.56 (0.55, 0.58)		0.67 (0.65, 0.70)
Anticoagulant Use	3822 (4.4%)		3558 (4.1%)	1.08 (1.03, 1.13)		0.94 (0.89, 0.99)
Case-control study predicting ICU admission/mechanical ventilation						
Statin Use	2111 (9.8%)		2379 (11.0%)	0.86 (0.81, 0.92)		0.90 (0.84, 0.97)
ACEI/ARB Use	2051 (9.5%)		2289 (10.6%)	0.88 (0.82, 0.94)		0.92 (0.86, 0.99)
Anticoagulant Use	1064 (4.9%)		1014 (4.7%)	1.05 (0.96, 1.15)		0.96 (0.88, 1.06)
Case-control study predicting hospital mortality						
Statin Use		164 (7.1%)	233 (10.1%)	0.66 (0.53, 0.82)	0.84 (0.65, 1.07)	
ACEI/ARB Use		152 (6.6%)	238 (10.3%)	0.60 (0.48, 0.74)	0.61 (0.47, 0.78)	
Anticoagulant Use		75 (3.3%)	135 (5.9%)	0.55 (0.41, 0.73)	0.56 (0.41, 0.77)	

⁎ Multivariable analyses were adjusted for all unmatched variables including statin use, ACEI/ARB use, anticoagulant use, ethnicity, region, and Elixhauser score.

⁎⁎ Each case-control study was matched on sex, race, age group, and each of the following medical conditions: COPD, obesity, cardiovascular disease, angina, diabetes, hyperlipidemia, and hypertension.

† OR, odds ratio.

‡ 95% CI, 95% confidence interval.

## Discussion

In this matched nested case-control study of a national cohort of patients diagnosed with COVID-19, we found that the use of statins, ACEI/ARBs, and anticoagulants was protective against a number of adverse COVID-19 outcomes. Using matching and multivariable adjustment, we accounted for multiple potentially confounding factors, including sex, age, race, ethnicity, region, and underlying medical conditions and medications. Moreover, our primary findings persisted across multiple sensitivity analyses, using exposure periods that extended to 60 and 120 days before the COVID-19 diagnosis and restricting to a cohort of patients treated in health systems with a high volume of COVID-19 patients.

It is likely that the observed protective effects on COVID-19 by statins are attributable, in some measure, to their antiinflammatory, antithrombotic, and immunomodulatory effects via a reduction in viral replication and suppression of the damaging effects of cytokine storm and microvascular and macrovascular thrombosis.^8^^,^^11^, ^12^, ^13^, ^14^ Zhang et al. showed an association between statin use and decreased risk of mortality and ventilator use, with lower levels of inflammatory markers among statin users.^8^ However, observational studies, randomized controlled trials, and metaanalyses have produced conflicting data on the utility of statin use for COVID-19 clinical outcomes.^8^^,^^11^^,^^38^, ^39^, ^40^, ^41^, ^42^, ^43^, ^44^, ^45^, ^46^ While a number of studies reported that the use of statins was associated with lower mortality rates and decreased inflammatory response in hospitalized COVID-19 patients,^8^^,^^39^^,^^41^^,^^42^^,^^44^ especially critically ill patients,^11^ other studies did not support the use of statins for hospitalized COVID-19 patients.^45^^,^^47^ Cariou et al. found that statin treatment was associated with increased mortality in a cohort of 2449 hospitalized patients with both type 2 diabetes mellitus and COVID-19,^38^ and one randomized controlled trial reported that atorvastatin use significantly increased the frequency of ICU admission and length of stay at the hospital for inpatients with COVID-19.^45^

The protective effects of ACEI/ARBs were possibly driven, to some degree, by antiinflammatory effects in COVID-19 patients,^15^^,^^16^ specifically by inhibiting ACE2 downregulation-induced hyperactivation of the RAAS caused by the SARS-CoV-2 virus.^7^ By attenuating the deleterious effects of angiotensin II, ACEI/ARBs may reduce oxidative stress, vasoconstriction, and inflammation in the lungs.^17^ Notably, there is concern that critical biases may have distorted the evidence in past observational research.^48^ Further, Bauer et al. reported that older, vulnerable patients might benefit from temporary discontinuation of RAAS inhibition for improved recovery from COVID-19.^49^

Given that coagulopathy—which commonly occurs as a result of COVID-19-related systemic thrombo-inflammation and results in a consequent risk of vascular permeability and hypercoagulation—is associated with increased disease severity and mortality, there has been interest in examining the effect of anticoagulants in COVID-19 patients. Postmortem examinations of COVID-19 patients showed thrombosis in pulmonary vessels and other organs without SARS-CoV-2 penetration.^27^^,^^50^ The observed protective effects of anticoagulants in this study were generally consistent with some past studies^28^^,^^29^^,^^31^^,^^51^ but inconsistent with others,^6^^,^^32^^,^^33^ including a randomized controlled trial by Connors et al. that was terminated early.^30^

Overall, our observation that the outpatient use of statins, ACEI/ARBs, and anticoagulants was protective against adverse outcomes in patients diagnosed with COVID-19 is consistent with a review conducted by Alves et al.^52^ and previous studies that assessed premorbid use of these classes of cardiovascular medications.^53^, ^54^, ^55^, ^56^, ^57^ It is important to note that ours is, to our knowledge, the largest matched, national comparative analysis of cardiovascular medications and COVID-19 outcomes thus far.

The results of our study may have been influenced by several limitations. First, medication use was defined by written prescriptions identified in the patient's EHR prior to COVID-19 diagnosis. It is possible that this record did not include information on over-the-counter (OTC) medications and thus contained no information on coadministration of these drugs. Many OTC medications, including aspirin and nonsteroidal antiinflammatory drugs, may influence the study outcomes and these data were not available in our database. Second, reliance on EHR data precluded assessment of a number of potential confounding factors such as diet, alcohol, medication adherence, lifestyle habits, prior state of health, and other health behaviors. Medication adherence to statins in particular has been recognized as a global challenge.^58^ Using EHR data also leads to a certain degree of misclassification by relying on codes in administrative claims data, but we attempted to minimize this by using standardized categorization algorithms, as well as multiple sensitivity analyses. Third, our data provide information on the date the medication was prescribed but not on the date it was filled, purchased, or picked up by the patient. It is possible, therefore, that some of the drug exposure periods used in this study underestimated the true medication exposure period. Our sensitivity analyses assessing 60- and 120-day exposure windows, however, help to address this issue.

Despite these limitations, this study has a number of strengths, including a large nationally representative sample, matching based on sociodemographic and disease risk factors, simultaneous adjustment for potentially confounding medical conditions and medications, and assessment of multiple exposure windows. This large, national matched comparative analysis of cardiovascular medications and COVID-19 outcomes addresses a critically important clinical and public health issue.

Our findings also suggest the need for a large randomized comparative effectiveness trial of statin, ACEI/ARB, and anticoagulant therapies alone or in combination as potential therapies for COVID-19 patients, regardless of history of ASCVDs. Other areas of study include examining comparative effectiveness of different statins—based on their lipophilic versus hydrophilic property—in COVID-19 patients as well as the effect of dose and duration with statins, ACEI/ARBs, and anticoagulants.

## Conclusion

Our study suggests that all three medications studied—statins, ACEI/ARBs, and anticoagulants—were associated with decreased risks of the adverse outcomes under study. Even with the increased availability of COVID-19 vaccines and treatments, immunocompromised and elderly patients with COVID-19 remain at risk for severe adverse outcomes. As of February 2023, COVID-19 is still a major contributor to premature deaths in the United States, with an approximately 21-day average of 452 new COVID-19 deaths per day.^59^ Our findings may help to guide clinical decision-making on choices of cardiovascular medications to continue or adjust for COVID-19 patients with multimorbidity and ASCVD.

## Role of the Sponsors

The funding organizations had no role in the design or conduct of the study; in the collection, analysis, or interpretation of data; or in the preparation, review, or approval of the manuscript.

## CRediT authorship contribution statement

**Leonard K. Wang:** Conceptualization, Writing – original draft, Writing – review & editing, Visualization. **Yong-Fang Kuo:** Conceptualization, Methodology, Formal analysis, Writing – original draft, Writing – review & editing, Supervision, Project administration, Funding acquisition. **Jordan Westra:** Formal analysis, Writing – original draft, Writing – review & editing, Visualization. **Mukaila A. Raji:** Conceptualization, Methodology, Formal analysis, Writing – original draft, Writing – review & editing, Funding acquisition. **Mohanad Albayyaa:** Writing – original draft, Writing – review & editing. **Joseph Allencherril:** Writing – original draft, Writing – review & editing. **Jacques Baillargeon:** Conceptualization, Methodology, Formal analysis, Writing – original draft, Writing – review & editing, Supervision, Project administration, Funding acquisition.

## Declaration of Competing Interest

The authors declare that they have no known competing financial interests or personal relationships that could have appeared to influence the work reported in this paper.

## Figures and Tables

**Appendix Table 4:** LOINC Codes for Positive COVID-19 Laboratory Test.

94306-8	SARS-CoV-2 RNA panel, unspecified specimen
94307-6	SARS-CoV-2 N gene, unspecified specimen
94308-4	SARS-CoV-2 N gene, unspecified specimen
94309-2	SARS-CoV-2 RNA, unspecified specimen
94311-8	SARS-CoV-2 N gene, unspecified specimen
94312-6	SARS-CoV-2 N gene, unspecified specimen
94314-2	SARS-CoV-2 RdRp gene, unspecified specimen
94316-7	SARS-CoV-2 N gene, unspecified specimen
94500-6	SARS-CoV-2 RNA, respiratory specimen
94503-0	SARS-CoV-2 IgG and IgM panel, serum or plasma
94504-8	SARS-CoV-2 IgG and IgM panel, serum or plasma
94505-5	SARS-CoV-2 IgG, serum or plasma
94506-3	SARS-CoV-2 IgM, serum or plasma
94507-1	SARS-CoV-2 IgG, serum or plasma
94508-9	SARS-CoV-2 IgM, serum or plasma
94510-5	SARS-CoV-2 N gene, unspecified specimen
94511-3	SARS-CoV-2 ORF1ab region, unspecified specimen
94531-1	SARS coronavirus 2 RNA panel, respiratory specimen
94533-7	SARS-CoV-2 N gene, respiratory specimen
94534-5	SARS-CoV-2 RdRp gene, respiratory specimen
94547-7	SARS-CoV-2 IgG+IgM, serum or plasma
94558-4	SARS-CoV-2 Ag, respiratory specimen
94559-2	SARS-CoV-2 ORF1ab region, respiratory specimen
94562-6	SARS-CoV-2 IgA, serum or plasma
94563-4	SARS-CoV-2 IgG, serum or plasma
94564-2	SARS-CoV-2 IgM, serum or plasma
94565-9	SARS-CoV-2 RNA, nasopharyngeal specimen
94639-2	SARS-CoV-2 ORF1ab region, unspecified specimen
94640-0	SARS-CoV-2 S gene, respiratory specimen
94641-8	SARS-CoV-2 S gene, unspecified specimen
94642-6	SARS-CoV-2 S gene, respiratory specimen
94643-4	SARS-CoV-2 S gene, unspecified specimen
94644-2	SARS-CoV-2 ORF1ab region, respiratory specimen
94645-9	SARS-CoV-2 RdRp gene, unspecified specimen
94646-7	SARS-CoV-2 RdRp gene, respiratory specimen
94660-8	SARS-CoV-2 RNA, serum/plasma
94661-6	SARS-CoV-2 antibody interpretation
94720-0	SARS-CoV-2 IgA, serum or plasma
94745-7	SARS-CoV-2 RNA, respiratory specimen
94746-5	SARS-CoV-2 RNA, unspecified specimen
94756-4	SARS-CoV-2 N gene, respiratory specimen
94757-2	SARS-CoV-2 N gene, respiratory specimen
94759-8	SARS-CoV-2 N overall result, nasopharyngeal specimen
94760-6	SARS-CoV-2 N gene result, nasopharyngeal specimen
94761-4	SARS-CoV-2 IgG Ab, DBS
94762-2	SARS-CoV-2 Ab qualitative
94763-0	SARS-CoV-2, unspecified specimen
94764-8	SARS-CoV-2 whole genome
94766-3	SARS-CoV-2 N gene, serum or plasma
94767-1	SARS-CoV-2 S gene, serum or plasma
94768-9	SARS-CoV-2 IgA, serum or plasma
94769-7	SARS-CoV-2 IgA, serum or plasma
94819-0	SARS-CoV-2 N gene, unspecified specimen
94822-4	SARS-CoV-2 RNA, saliva
94845-5	SARS-CoV-2 RNA, saliva
95125-1	SARS-CoV-2 RNA, serum or plasma
95406-5	SARS-CoV-2 RNA, nose
95409-9	SARS-CoV-2 N gene, nose
95410-7	SARS-CoV-2 neutralizing antibody, serum
95411-5	SARS-CoV-2 neutralizing antibody, serum
95416-4	SARS-CoV-2 IgM Ab, DBS
95424-8	SARS-CoV-2 RNA, respiratory specimen
95425-5	SARS-CoV-2 N gene, saliva
95427-1	SARS-CoV-2 IgA Ab, serum or plasma
95428-9	SARS-CoV-2 IgM Ab, serum or plasma
95429-7	SARS-CoV-2 IgG Ab, serum or plasma
95521-1	SARS-CoV-2 N gene, respiratory specimen
95522-9	SARS-CoV-2 N gene, respiratory specimen
